# Supplementary material for: Mechanism of pore opening in the calcium-activated chloride channel TMEM16A
Source: Nat Commun. 2021 Feb 4;12:786. doi: 10.1038/s41467-020-20788-8 (PMC7862263; doi:10.1038/s41467-020-20788-8)
Supplement: Supplementary file 1 — Supplementary Information [file 41467_2020_20788_MOESM1_ESM.pdf]

Supplementary Information

Mechanism of pore opening in the calcium-activated chloride channel TMEM16A

Andy K. M. Lam and Raimund Dutzler

Supplementary Figures

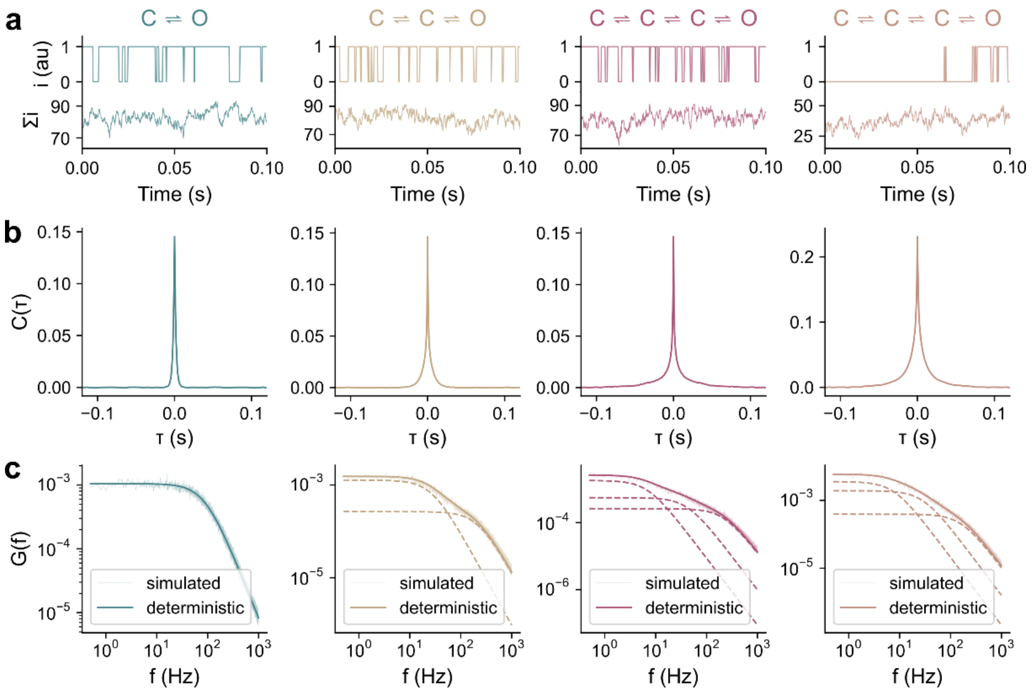

Supplementary Fig. 1: Relation between mechanism, autocorrelation function, and power spectrum. a, Representative sections of single-channel trajectories and the corresponding ensemble currents simulated according to the indicated mechanisms using an approximation of a stochastic simulation algorithm. The ensemble current is the sum of 100 simulated single-channel trajectories with a unitary amplitude of 1 a.u. **b**, Averaged autocorrelation functions and **c**, power spectra of the simulated single-channel time series overlaid with the corresponding deterministic solutions. Dashed lines indicate the underlying Lorentzian components.

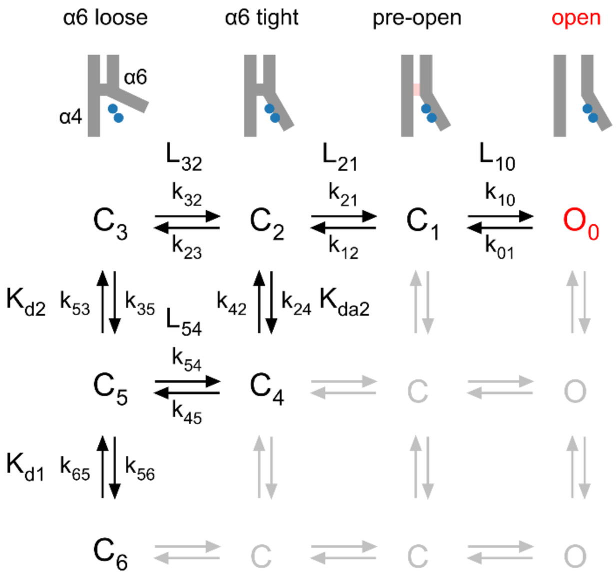

Supplementary Fig. 2: Kinetic model of TMEM16A activation. Minimal gating model used to analyze the power spectra of TMEM16A at different Ca^{2+} concentrations. Schematic view of conformational states is depicted at the top, the states that are included in the model are shown in black, and states omitted from the fit are depicted in grey. Rate and equilibrium constants connecting pairs of states are indicated.

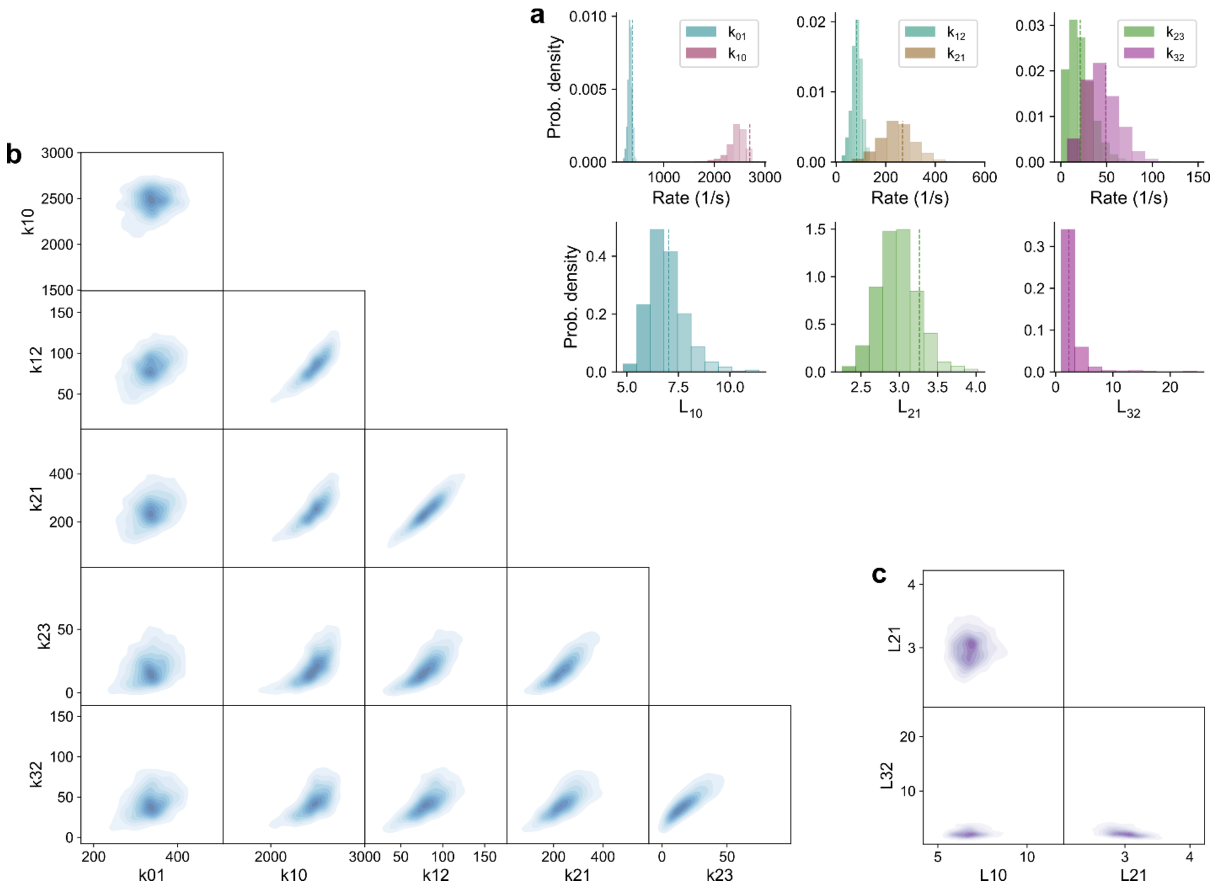

Supplementary Fig. 3: Monte Carlo confidence intervals and parameter distributions. a, Histograms of parameters estimated from power spectra calculated from 1,000 simulated single-channel records. Dashed lines indicate the true values used in the simulations. (**b** and **c**) Pairwise correlation between the best-fit values for **b**, the individual rate constants and **c**, equilibrium constants.

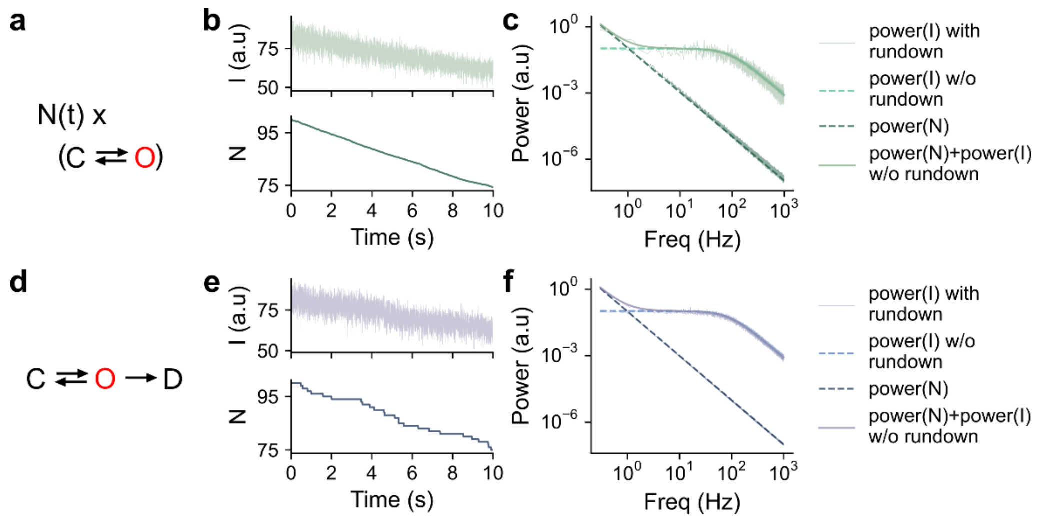

Supplementary Fig. 4: Effect of non-stationarity. a, Mechanism depicting time-dependent decrease in the number of activatable channels as observed during current rundown in TMEM16A. **b**, Ensemble current (top, I) and number of surviving channels (bottom, N) simulated using an approximation of a stochastic simulation algorithm using the model shown in (**a**). **c**, Power spectra of the monoexponentially decaying ensemble current, that due to channel fluctuations alone, and that corresponding to the decay in channel number. (**d**–**f**) Same as (**a**–**c**) but with a mechanism depicting time-dependent decrease in current due to slow, pseudo-irreversible entry into a desensitized state. (**c** and **f**) Lines are deterministic solutions of the fluctuations expected for the mechanism (Eq. 16), that describing the autocorrelation of the exponential decrease in the channel number (Eq. 40), and their sum.

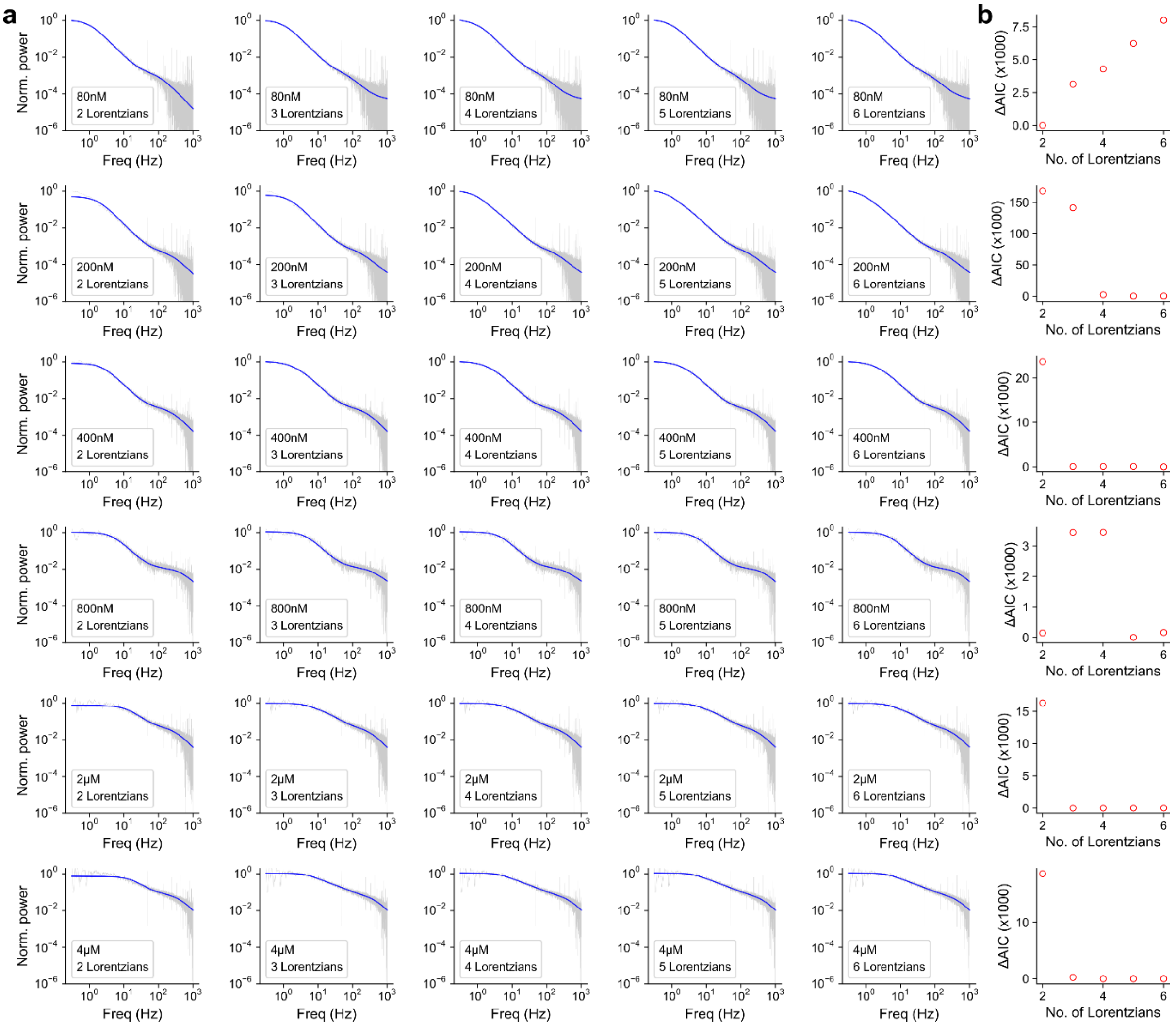

Supplementary Fig. 5: Assessment of the number of Lorentzian components. a, Power spectra from Fig. 1b fitted with the indicated number of Lorentzian components. Solid lines are the best-fit. **b**, Difference Akaike's information criterion (ΔAIC) for the corresponding fits.

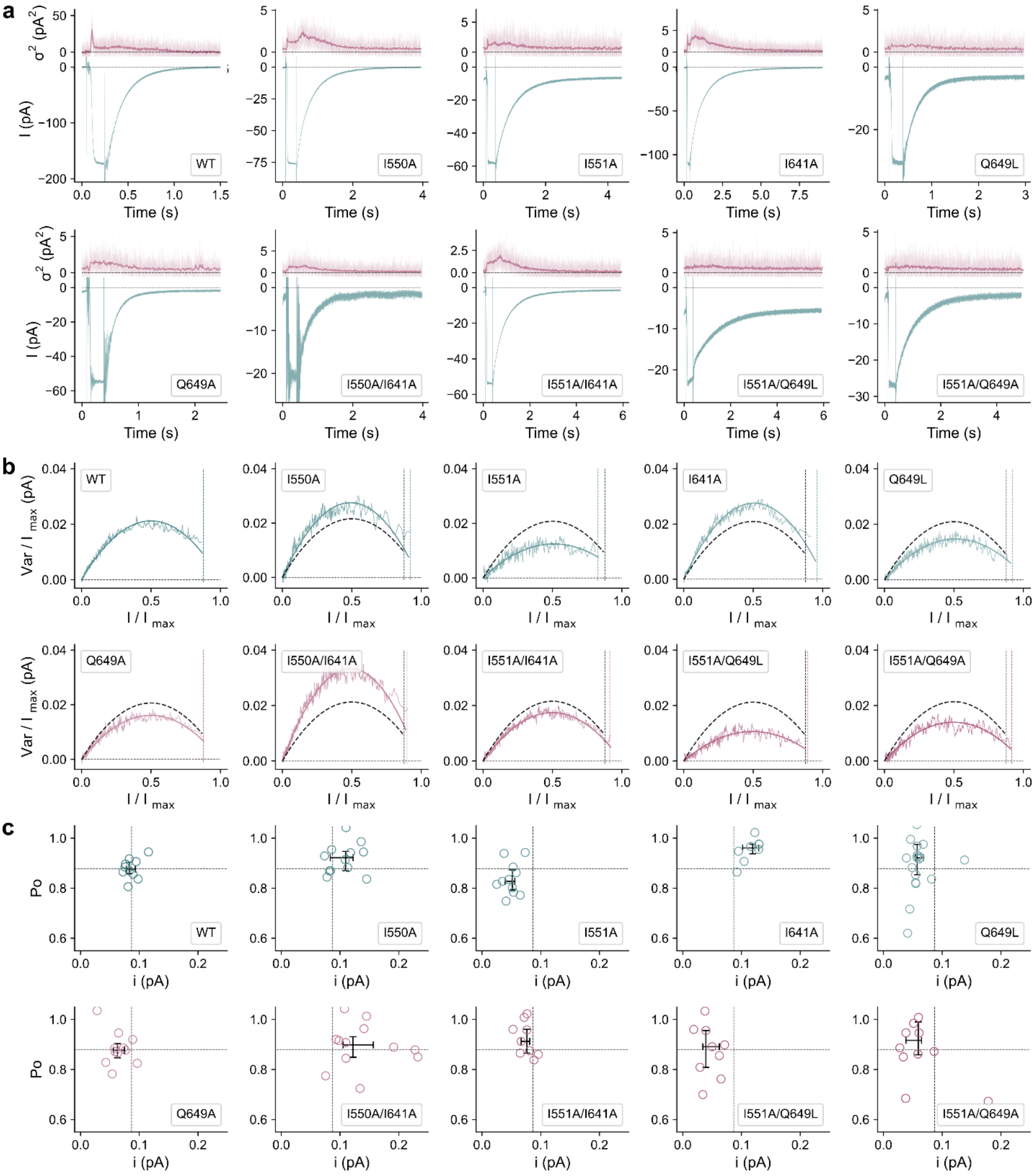

Supplementary Fig. 6: Non-stationary noise analysis. a, Representative mean current and the calculated variance upon a step-exchange from zero Ca^{2+} to a saturating Ca^{2+} concentration and back. **b**, Merged and averaged variance-mean current relations at saturating Ca^{2+} concentrations. Data are averages of the indicated number of patches (WT, n=10; I550A, n=12; I551A, n=11; I641A, n=8; Q649L, n=21; Q649A, n=11; I550A/I641A, n=12; I551A/I641A, n=9; I551A/Q649L, n=9; I551A/Q649A, n=12). Solid lines are fits to Eq. 3. Dashed lines correspond to the relation of WT. Dotted lines indicate the median of the respective estimated Po. **c**, Two-dimensional distributions of the estimated single-channel current (i) and open probability (Po). The black cross indicates the median and interquartile range for the estimated i and Po values. The crossing of the dotted lines indicates the median values of WT.

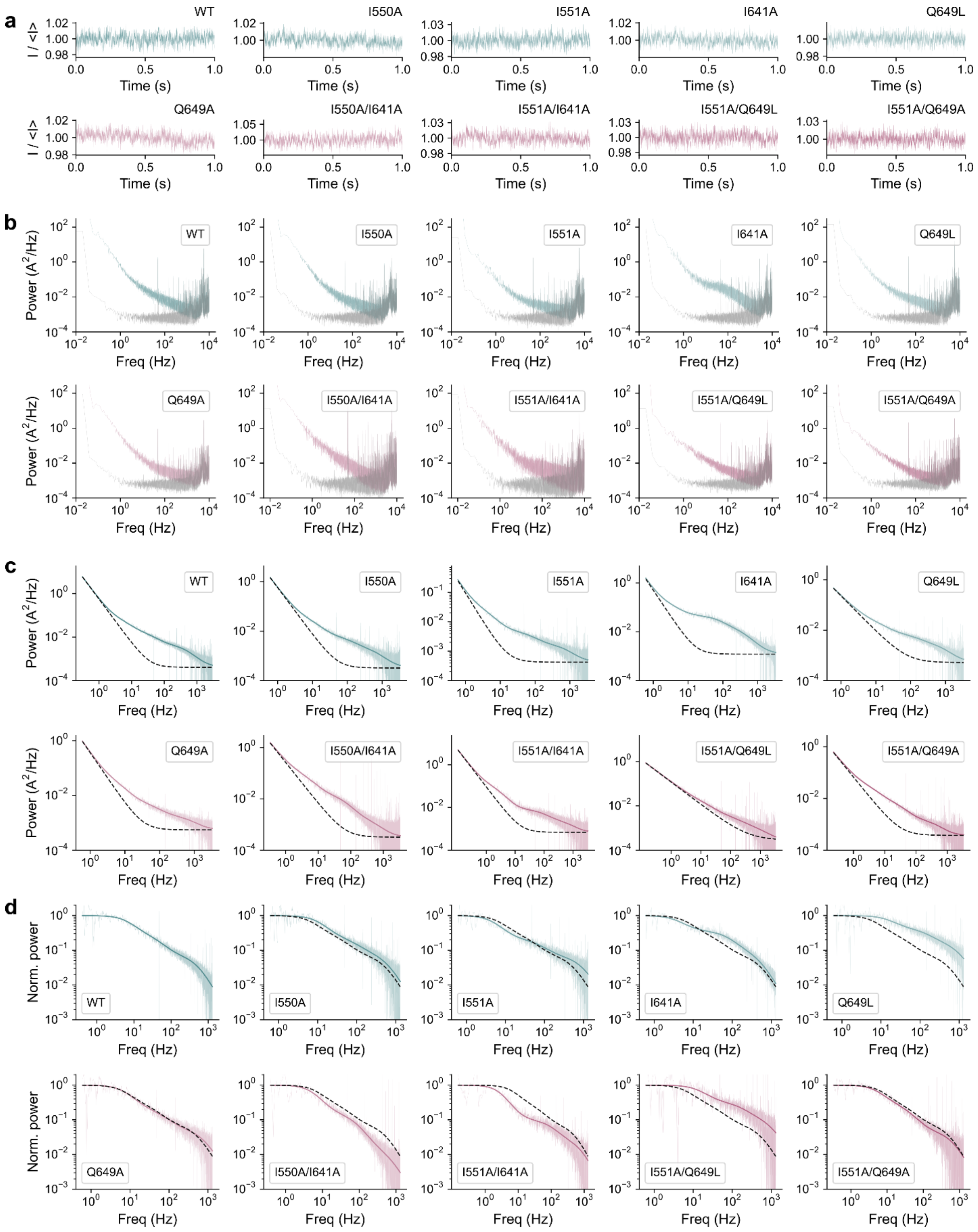

Supplementary Fig. 7: Spectral analysis of gate mutants at saturating Ca^{2+} concentrations. **a**, Representative section of steady-state currents at saturating Ca^{2+} concentrations. For display, the current traces were filtered at 2 kHz using a digital 4-pole Bessel lowpass filter. **b**, Raw power spectra calculated from the steady-state currents shown

in (**a**) and the corresponding background recorded at the reversal potential of Cl^- (grey). **c**, Background-subtracted power spectra. Solid lines are empirical fits to Eq. 6 comprising the 1/f and constant components, and components corresponding to gating-induced fluctuations. Dashed lines correspond to the estimated 1/f and constant components that are subtracted from the total spectrum to yield the gating component. **d**, Normalized power spectra reflecting the fluctuations due to gating. Solid lines are fits to Eq. 16. Dashed line corresponds to the wild-type spectrum. (**b–d**) Data are averages of the indicated number of patches (WT, n=7; I550A, n=6; I551A, n=7; I641A, n=7; Q649L, n=7; Q649A, n=7; I550A/I641A, n=7; I551A/I641A, n=6; I551A/Q649L, n=7; I551A/Q649A, n=8).

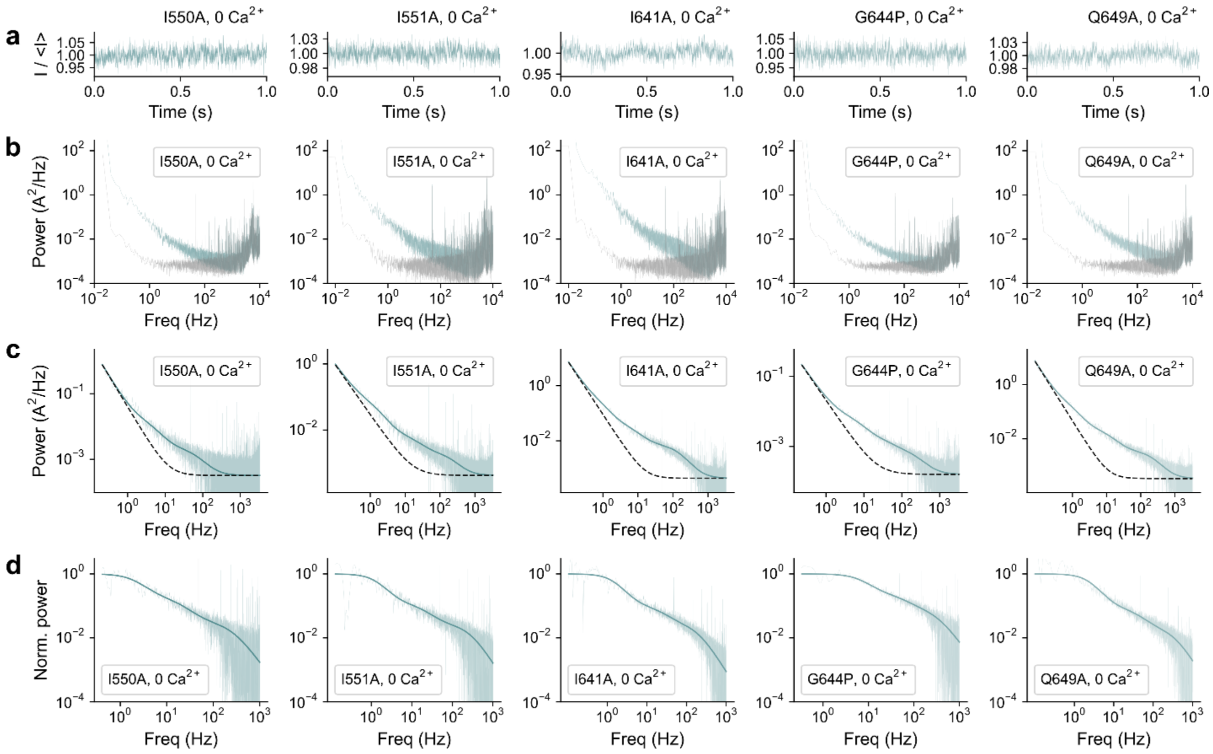

Supplementary Fig. 8: Spectral analysis of selected mutants at zero Ca^{2+}. a, Representative section of steady-state currents at zero Ca^{2+}. For display, the current traces were filtered at 2 kHz using a digital 4-pole Bessel lowpass filter. **b**, Raw power spectra calculated from the steady-state currents shown in (**a**) and the corresponding background recorded at the reversal potential of Cl^- (grey). **c**, Background-subtracted power spectra. Solid lines are empirical fits to Eq. 6 comprising the 1/f and constant components, and components corresponding to gating-induced fluctuations. Dashed lines correspond to the estimated 1/f and constant components that are subtracted from the total spectrum to yield the gating component. **d**, Normalized power spectra reflecting the fluctuations due to gating. Solid lines are fits to Eq. 16. (**b–d**) Data are averages of the indicated number of patches (I550A, n=6; I551A, n=7; I641A, n=7; G644P, n=9; Q649A, n=7).

Supplementary Tables

Supplementary Table 1: Fitted parameters for WT in a full mechanism with Ca^{2+} binding steps explicitly included (using Po^{max} at +80 mV).

	Unit	Best-fit	95% CI	95% CI / Best-fit
Rate constants				
k_{01}	s^{-1}	306	3.3	0.01
k_{10}	s^{-1}	1967	565.0	0.3
k_{12}	s^{-1}	73	2.7	0.04
k_{21}	s^{-1}	288	10.4	0.04
k_{23}	s^{-1}	17	2.9	0.2
k_{32}	s^{-1}	34	5.1	0.2
k_{24}	s^{-1}	437	868.6	2.0
k_{42}	$M\ s^{-1}$	12,244,810,972	655,365,755	0.1
k_{35}	s^{-1}	25	6.6	0.3
k_{53}	$M\ s^{-1}$	20,662,103	5,432,785	0.3
k_{45}	s^{-1}	24,201	23,428	1.0
k_{54}	s^{-1}	1405	181.5	0.1
k_{56}	s^{-1}	454,072	25,846.4	0.1
k_{65}	$M\ s^{-1}$	1E+11	n/a	n/a
Equilibrium constants				
L_{10}		6.4	1.9	0.3
L_{21}		3.9	0.2	0.05
L_{32}		2.0	0.5	0.2
L_{54}		0.06	0.06	1.0
L_{56} (K_{d1})	µM	4.5	0.3	0.1
L_{35} (K_{d2})	µM	1.2	0.5	0.4
L_{24} (K_{da2})	µM	0.04	n/a	n/a

k_{65} was assumed to be diffusion-limited. K_{da2} was assumed to represent the highest affinity in the system, which was experimentally determined in an accompanying manuscript[13]. n/a, not applicable; CI, confidence interval.

Supplementary Table 2: Fitted parameters for the indicated constructs at saturation (using Po^{max} at –40 mV).

	WT		I550A		I551A		I641A		I550A/I641A	
[Ca^{2+}]	4 µM		2 µM		2 µM		2 µM		2 µM	
	Best-fit	95% CI	Best-fit	95% CI	Best-fit	95% CI	Best-fit	95% CI	Best-fit	95% CI
k_{01} (s^{-1})	275	2.6	193.9	4.4	774	17.1	78.7	0.9	292.4	74.3
k_{10} (s^{-1})	2,894	832	3,188	477.5	4,723	466.5	2,693	146.5	5,071.2	1,759.1
k_{12} (s^{-1})	118	9.3	140	12.5	96	5.2	188.8	5.8	274.8	43.0
k_{21} (s^{-1})	407	36.2	565	36.6	495	17.7	561.9	7.9	409.1	35.7
k_{23} (s^{-1})	38	6.7	51.4	2.4	14.3	0.5	4.4	0.1	14.7	1.5
k_{32} (s^{-1})	60	6.1	80.4	1.3	34.8	0.6	27.3	0.4	34.3	1.5
L_{10}	10.5	3.0	16.4	2.5	6.1	0.6	34.2	1.9	17.3	7.4
L_{21}	3.4	0.4	4	0.5	5.1	0.3	3	0.1	1.5	0.3
L_{32}	1.6	0.3	1.6	0.1	2.4	0.1	6.1	0.2	2.3	0.3
	Mean	SEM	Mean	SEM	Mean	SEM	Mean	SEM	Mean	SEM
Po	0.88	0.01	0.92	0.02	0.84	0.02	0.95	0.02	0.89	0.03

	I551A/I641A		Q649L		Q649A		I551A/Q649L		I551A/Q649A	
[Ca^{2+}]	2 µM		2 µM		2 µM		2 µM		2 µM	
	Best-fit	95% CI	Best-fit	95% CI	Best-fit	95% CI	Best-fit	95% CI	Best-fit	95% CI
k_{01} (s^{-1})	288.6	8.8	369.8	9.4	411.8	8.3	494.1	56.4	227.8	14.5
k_{10} (s^{-1})	4,328.5	426.0	5, 454.7	855.1	4,130	350.5	5,417.5	2851.5	3,645.4	811.5
k_{12} (s^{-1})	203.2	12.6	199.5	16.6	108.6	5.0	300.6	98.0	81.9	7.72
k_{21} (s^{-1})	680.7	20.9	991.1	50.0	421.4	15.1	1,182.5	199.4	243.2	18.5
k_{23} (s^{-1})	9.4	0.3	31.1	2.0	31.8	1.0	35	6.2	14.2	1.6
k_{32} (s^{-1})	19.6	0.2	114.5	3.7	54.5	0.7	102.1	7.9	38.9	2.0
L_{10}	15	1.6	14.8	2.3	10	0.9	11	5.9	16	3.7
L_{21}	3.4	0.2	5	0.5	3.9	0.2	3.9	1.4	3	0.6
L_{32}	2.1	0.07	3.7	0.3	1.7	0.06	2.9	0.6	2.7	0.3
	Mean	SEM	Mean	SEM	Mean	SEM	Mean	SEM	Mean	SEM
Po	0.92	0.02	0.92	0.03	0.88	0.02	0.87	0.03	0.91	0.04

CI, confidence interval.

Supplementary Table 3: Fitted parameters for the indicated constructs at zero Ca^{2+} (using Po estimated from Po^{max} and unitary current ratio, see Methods).

	I550A		I551A		I641A		G644P		Q649A	
$[Ca^{2+}]$	0		0		0		0		0	
	Best-fit	95% CI	Best-fit	95% CI	Best-fit	95% CI	Best-fit	95% CI	Best-fit	95% CI
k_{01} (s^{-1})	455	139.77	539.94	25.59	322.98	6.56	305.64	11.5	510.61	14.29
k_{10} (s^{-1})	1064.7	855.18	1077.1	177.4	810.9	350.2	1608.4	396.2	1509.6	446.9
k_{12} (s^{-1})	64.77	29.23	46.74	4.8	59.43	8.57	114.68	17.85	116.12	12.86
k_{21} (s^{-1})	107.06	35.92	116.12	8.19	142.27	21.28	358.76	38.09	241.21	25.23
k_{23} (s^{-1})	9.03	2.99	6.91	0.35	10.02	2.49	32.57	2.39	17.5	2.81
k_{32} (s^{-1})	13.2	2.05	8.28	0.18	9.05	1.75	48.56	1.27	14.76	2.06
L_{10}	2.34	2.01	1.99	0.34	2.51	1.09	5.26	1.31	2.96	0.88
L_{21}	1.65	0.91	2.48	0.31	2.39	0.5	3.13	0.59	2.08	0.32
L_{32}	1.46	0.53	1.2	0.07	0.9	0.28	1.49	0.12	0.84	0.18
	Mean	SEM	Mean	SEM	Mean	SEM	Mean	SEM	Mean	SEM
Po	0.54	0.19	0.53	0.03	0.57	0.06	0.77	0.21	0.59	0.05

CI, confidence interval.

Supplementary Table 4: List of primers.

Name	F/R	Sequence (5'-to-3')
I550A	F	GTG GTC GCC ATT CTG CTG GAT GAA GTT TAC GGC TGC ATT
I550A	R	CAG AAT GGC GAC CAC GAG GTT GAT GAT AAC AGC GGT GGC
I551A	F	GTC ATC GCC CTG CTG GAT GAA GTT TAC GGC TGC ATT GCC
I551A	R	CAG CAG GGC GAT GAC CAC GAG GTT GAT GAT AAC AGC GGT
I641A	F	AGC ATC GCC ATG CTG GGC AAG CAG CTA ATC CAG AAC AAT
I641A	R	CAG CAT GGC GAT GCT CAG CTG GAT ACA GAG CTC CAT GAG
G644P	F	ATG CTG CCG AAG CAG CTA ATC CAG AAC AAT CTC TTC GAG
G644P	R	CTG CTT CGG CAG CAT AAT GAT GCT CAG CTG GAT ACA GAG
Q649A	F	CTA ATC GCA AAC AAT CTC TTC GAG ATT GGC ATC CCG AAG
Q649A	R	ATT GTT TGC GAT TAG CTG CTT GCC CAG CAT AAT GAT GCT
Q649L	F	CTA ATC CTG AAC AAT CTC TTC GAG ATT GGC ATC CCG AAG
Q649L	R	ATT GTT CAG GAT TAG CTG CTT GCC CAG CAT AAT GAT GCT

F, forward; R, reverse
